# Increased fetal adiposity prior to diagnosis of gestational diabetes in South Asians: more evidence for the ‘thin–fat’ baby

**DOI:** 10.1007/s00125-016-4166-2

**Published:** 2016-12-02

**Authors:** Hema Venkataraman, Uma Ram, Sam Craik, Anuradhai Arungunasekaran, Suresh Seshadri, Ponnusamy Saravanan

**Affiliations:** 1 Populations, Evidence and Technologies, Division of Health Sciences, Warwick Medical School, University of Warwick, Gibbet Hill, Coventry, CV4 7AL UK; 2 Seethapathy Clinic and Hospital, Chennai, India; 3 Mediscan Systems, Mylapore, Chennai, India; 4 Department of Diabetes, Endocrinology & Metabolism, George Eliot Hospital, Nuneaton, UK

**Keywords:** Abdominal fat, Fetal adiposity, Gestational diabetes, Intrauterine programming, Offspring metabolic risk, Prediction

## Abstract

**Aims/hypothesis:**

Gestational diabetes mellitus (GDM) is associated with an increased future risk of obesity in the offspring. Increased adiposity has been observed in the newborns of women with GDM. Our aim was to examine early fetal adiposity in women with GDM.

**Methods:**

Obstetric and sonographic data was collated for 153 women with GDM and 178 controls from a single centre in Chennai, India. Fetal head circumference (HC), abdominal circumference (AC), femur length (FL) and biparietal diameter (BPD) were recorded at 11, 20 and 32 weeks. Anterior abdominal wall thickness (AAWT) as a marker of abdominal adiposity at 20 and 32 weeks was compared between groups. Adjustments were made for maternal age, BMI, parity, gestational weight gain, fetal sex and gestational age.

**Results:**

Fetuses of women with GDM had significantly higher AAWT at 20 weeks (β 0.26 [95% CI 0.15, 0.37] mm, *p* < 0.0001) despite lower measures of HC, FL, BPD and AC. AAWT remained higher in the fetuses of women with GDM at 32 weeks (β 0.48 [0.30, 0.65] mm, *p* < 0.0001) despite similar measures for HC, FL, BPD and AC between groups. Both groups had similar birthweights at term. There was an independent relationship between fasting plasma glucose levels and AAWT after adjustment as described above.

**Conclusions/interpretation:**

A ‘thin but fat’ phenotype signifying a disproportionate increase in adiposity despite smaller or similar lean body mass was observed in the fetuses of mothers with GDM, even at 20 weeks, thus pre-dating the biochemical diagnosis of GDM. Increased AAWT may serve as an early marker of GDM.

**Electronic supplementary material:**

The online version of this article (doi:10.1007/s00125-016-4166-2) contains peer-reviewed but unedited supplementary material, which is available to authorised users.

## Introduction

Gestational diabetes mellitus (GDM) is associated with several maternal and neonatal complications [1]. Increased fetal size, defined as macrosomia or large for gestational age (LGA), is an important early fetal complication, which has been shown to be reduced by up to 50% with intervention [2, 3]. In the long term, the offspring of mothers with GDM have a two- to fourfold higher future risk of obesity and diabetes [4–6], with evidence for increased BMI, body fat and subcutaneous abdominal fat in early childhood and adolescence [7–9].

There is emerging evidence that this increase in adiposity in the offspring of women with GDM begins in early fetal life [10, 11]. Such fetuses exhibit a ‘thin–fat’ phenotype i.e. have preferential growth of insulin sensitive adipose tissue mass over that of fat-free lean tissues [10], with a higher total fat:lean mass ratio [12]. However, current evidence for a link between increased fetal adiposity and GDM is restricted to late pregnancy [11–14] and birth [10, 15, 16] (electronic supplementary material [ESM] Table 1).

Recently, Sovio et al reported early growth differences in the fetuses of women with GDM and controls in a prospective cohort of nulliparous women in the UK [17]. Using abdominal circumference (AC) as a surrogate for fetal adiposity, they observed increased fetal AC for women with GDM even at the time of diagnosis (at 28 weeks), although no difference was observed between groups at 20 weeks. In addition, the AC growth velocity at 20–28 weeks was significantly higher in the fetuses of mothers who later developed GDM. Traditionally, GDM is diagnosed at around 28 weeks of gestation by an OGTT [18]. Such evidence for early abnormal fetal growth pre-dating the routine biochemical diagnosis of GDM, calls for an earlier diagnosis and intervention strategy for GDM.

Anterior abdominal wall thickness (AAWT) has been used previously as a reliable marker of fetal abdominal adiposity [10, 14, 19]. Our aim was to assess fetal body size and abdominal adiposity in GDM during early pregnancy, prior to the biochemical diagnosis of GDM.

## Methods

A retrospective analysis of maternal demographic, anthropometric and fetal sonographic data was conducted for all women attending the Seethapathy Clinic & Hospital, Chennai, India, for routine antenatal care from September 2011 to December 2013. Patient consent and investigations were carried out in accordance with the revised Declaration of Helsinki (2008) (www.wma.net/en/30publications/10policies/b3/index.html).

A total of 153 consecutive women diagnosed as having GDM at the time of an OGTT (24–28 weeks) were identified. For each patient, at least one age- and parity-matched woman without GDM who was cared for during the same period and delivered in the same hospital was selected as a control. Women with, pre-existing diabetes, multiple pregnancy or fetal anomalies were excluded.

**Fig. 1:**
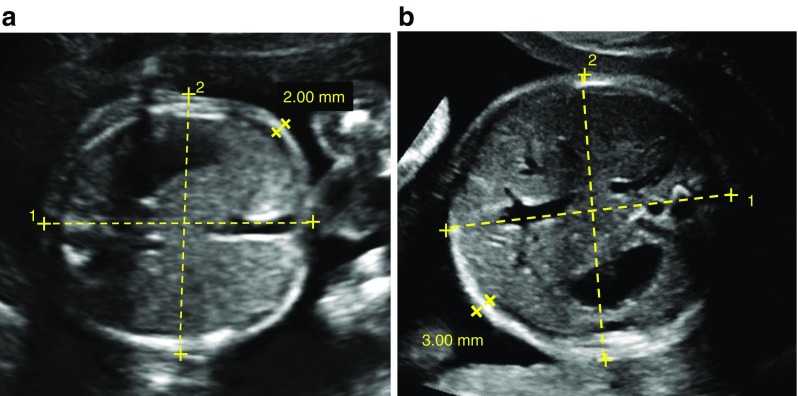
(**a**, **b**) AAWT measurements for (**a**) control and (**b**) GDM fetuses at 20 weeks of gestation. Yellow dashed lines 1 and 2 indicate the AC measurements. The space between the two bold X symbols at the edge of the abdominal wall indicates the AAWT

Ultrasound scans were performed at two centres, Mediscan and Seethapathy Hospital, by two independent sonographers using standardised techniques. All patients underwent a dating scan at 10–12 weeks and a fetal anomaly scan at 19–21 weeks. Women with GDM underwent fetal growth scans at 28, 32 and (if clinically indicated) 36 weeks of gestation. All control pregnant women had a scan at around 32 weeks, according to routine clinical practice in the region. Head circumference (HC), AC, femur length (FL) and biparietal diameter (BPD) were measured at each time point. AAWT was measured from archived images using SonoCare Medialogic Solutions software (version 6.8) by the two sonographers, who were blinded to disease status. AAWT measurements were obtained at 20 and 32 weeks using standardised techniques, as described previously [19] and used as measures of fetal adiposity. In brief, the standard plane used to measure the AC was utilised to calculate the AAWT. The AAWT was calculated as the thickness of the echogenic rim measured at a point 2–3 cm lateral to the umbilical cord insertion into the portion of the abdominal wall closer to the probe, taking care not to include the hypoechoic area between the abdominal wall and the liver (shown in Fig. 1).

Routinely recorded neonatal anthropometry consisting of HC, length and birthweight was retrieved. LGA was defined as birthweight >90th centile for gestational age, and small for gestational age (SGA) as birthweight <10th centile for gestational age, for gestational age ranging between 28 and 41 weeks. Birthweight centiles were calculated using the WHO weight percentiles calculator with a mean birthweight of 3230 g (SD 427.3 g) [20–24].

Gestational weight gain (GWG) was defined as the total weight gain between the first booking visit and delivery. The rate of GWG was calculated as the GWG divided by the number of weeks between booking and delivery. Since gestational age at booking was variable between participants, the rate of GWG was used instead of GWG as a covariate in regression models. Multiparity was defined as more than one live or still-birth after 24 weeks’ gestation.

### Diagnosis of GDM

Universal screening was used to diagnose GDM. All women underwent a fasting plasma glucose (FPG) test at booking. Those with FPG levels ≥7.0 mmol/l (126 mg/dl) were considered to have pre-gestational diabetes. Women with FPG levels of 5.1–6.9 mmol/l (92–125 mg/dl) at booking were considered to have GDM after confirmation by a second FPG test. For all other women, a 75 g OGTT was carried out at between 24 and 28 weeks. Only women who underwent an OGTT were included in this analysis. GDM was diagnosed based on the International Association of the Diabetes and Pregnancy Study Groups criteria i.e. a FPG level of ≥5.1 mmol/l (92 mg/dl), a 1 h plasma glucose (1hPG) level of ≥10.0 mmol/l (180 mg/dl) or a 2 h plasma glucose (2hPG) level of ≥8.5 mmol/l (153 mg/dl). A FPG level of <5.6 mmol/l (100 mg/dl) and a 1hPG level of <7.8 mmol/l (140 mg/dl) were considered the optimum glycaemic targets in pregnancy. Diet and lifestyle advice was given first to all women with GDM, followed by insulin if glycaemic targets were not met.

### Statistical analysis

The Student’s *t* test and χ^2^ test were used to compare means and proportions between the GDM and control groups. Multivariable linear regression was used to analyse differences in fetal biometric variables after adjustment for maternal age, BMI, parity, GWG, fetal sex and gestational age at scan. IBM SPSS Statistics software (version 22.0, Chicago, IL, USA) was used for all analyses.

## Results

A total of 178 controls and 153 women with GDM who had complete sonographic data were included in the analysis. Fetal biometry was compared between the GDM and control groups at 12, 20 and 32 weeks of gestation. Complete fetal biometric data was available for 325 women at the 20 week scan (mean ± SD, 20.9 ± 1.1 weeks) and for 316 women at the 32 week scan (mean ± SD, 32.5 ± 1.6 weeks). Archived images were unavailable for measuring AAWT for three women at the 20 week scan. Baseline characteristics of the two groups are shown in Table 1. While maternal age, BMI, height and parity were comparable between the two groups, women with GDM had significantly less GWG than controls. At birth, neonatal weight, HC and length, as well as the proportion of LGA and SGA neonates, were similar in the two groups.

**Table 1:** Baseline maternal and birth characteristics in the GDM and control groups

Characteristic	GDM group	Control group	*p* value
Maternal			
Age	28.5 ± 3.8	28.8 ± 4.2	0.610
BMI, kg/cm^2^	25.9 ± 5.8	23.7 ± 6.6	0.002
Height, cm	155.2 ± 19.1	150.8 ± 33.40	0.142
Weight, kg	65.9 ± 12.5	60.6 ± 16.0	0.001
Multiparity	7/145 (4.8)	3/175 (1.7)	0.123
Previous GDM	6/153 (3.9)	0/178 (0)	0.008
Family history of diabetes	75/153 (49.0)	59/178 (33.1)	0.003
FPG, mmol/l	5.3 ± 0.7	4.4 ± 0.5	<0.0001
1hPG, mmol/l	9.7 ± 1.9	7.0 ± 1.5	<0.0001
2hPG, mmol/l	8.1 ± 1.8	5.9 ± 1.2	<0.0001
GWG, kg	8.62 ± 4.34	10.73 ± 4.48	<0.0001
GWG/week, kg	0.34 ± 0.20	0.41 ± 0.17	0.001
Birth			
Male sex	69/153 (45.1)	98/178 (55.1)	0.071
Birthweight, g	3020 ± 464	3110 ± 383	0.070
Gestational age at birth, weeks	38.5 ± 1.5	38.8 ± 1.1	0.056
Caesarean section	83/153 (54.2)	75/178 (42.1)	0.028
Elective Caesarean section	26/83 (31.3)	27/75 (36.0)	0.534
HC, cm	33.75 ± 1.86	33.74 ± 1.36	1.00
Length, cm	47.79 ± 2.70	47.64 ± 2.14	0.61
LGA	35/153 (22.9)	42/178 (23.6)	0.877
SGA	21/153 (13.7)	15/178 (8.4)	0.123

Data are means ± SD or *n* (%)

*p* value refers to the Student’s *t* test for linear variables and the χ^2^ test for percentages

At the 12 week scan, both groups had similar fetal variables (GDM vs control group (mm): HC, 66.2 ± 28.7 vs 65.5 ± 29.6; AC, 52.3 ± 25.1 vs 51.7 ± 22.9; FL, 7.5 ± 7.3 vs 7.5 ± 7.5; BPD, 18.5 ± 7.9 vs 19.2 ± 9.9; *p* > 0.05 for all comparisons)

### Differences in fetal biometry at 20 weeks of gestation

The mean gestation at the 20 week scan was 20.9 ± 1.1 weeks (GDM vs control group: 21.06 ± 1.23 vs 20.82 ± 0.96, *p* = 0.062). Table 2 shows the differences in fetal biometry and AAWT between the GDM and control groups. Fetuses of women who were later diagnosed with GDM had a significantly higher AAWT but smaller measures for HC, AC, FL and BPD. These differences in traditional biometric measures and AAWT between the two groups persisted even after adjusting for maternal age, BMI, parity, GWG, fetal sex and gestational age at scan.

**Table 2:** Fetal biometric variables in the GDM and control groups

Fetal variable	GDM group (mean ± SD)	Control group (mean ± SD)	Adjusted β coefficient (95% CI)	*p* value
At 20 weeks’ gestation (prior to GDM diagnosis)				
HC, mm	178.12 ± 11.59	181.39 ± 11.21	−4.19 (−6.12, −2.26)	<0.0001
			−4.43 (−6.63, −2.23)^a^	<0.0001^a^
AC, mm	154.41 ± 11.53	156.57 ± 11.12	−2.99 (−5.03, −1.16)	0.004
			−2.63 (−4.92, −0.34)^a^	0.025^a^
FL, mm	34.29 ± 4.32	34.86 ± 2.77	−1.13 (−1.61, −0.65)	<0.0001
			−1.13 (−1.67, −0.58)^a^	<0.0001^a^
BPD, mm	48.92 ± 3.12	49.95 ± 3.31	−1.20 (−1.78, −0.62)	<0.0001
			−1.29 (−1.91, −0.63)^a^	<0.0001^a^
AAWT, mm	2.63 ± 0.51	2.39 ± 0.41	0.28 (0.17, 0.38)	<0.0001
			0.26 (0.15, 0.37)^a^	<0.0001^a^
At 28–32 weeks’ gestation				
HC, mm	288.23 ± 17.71	297.81 ± 14.15	−2.62 (−5.19, −0.06)	0.040
			−2.51 (−5.41, 0.38)^a^	0.089^a^
AC, mm	265.87 ± 22.04	276.78 ± 19.02	−2.82 (−6.21, 0.57)	0.100
			−2.09 (−5.86, 1.68)^a^	0.276^a^
FL, mm	60.14 ± 4.80	62.79 ± 3.85	−0.87 (−1.57, −0.16)	0.017
			−0.55 (−1.27, 0.17)^a^	0.131^a^
BPD, mm	80.73 ± 5.23	83.29 ± 4.42	−0.35 (−1.12, 0.42)	0.37
			−0.20 (−1.04, 0.65)^a^	0.65^a^
AAWT, mm	4.65 ± 0.81	4.37 ± 0.66	0.50 (0.34, 0.66)	<0.0001
			0.48 (0.30, 0.65)^a^	<0.0001^a^

The control group was used as the reference category

β coefficient for respective fetal biometry variables using multivariable linear regression after adjustment for maternal age, BMI, parity, fetal sex and gestational age

^a^Adjustment as above plus GWG/week

### Differences in fetal biometry at 32 weeks of gestation

The mean gestation at the 32 week scan was 32.5 ± 1.6 weeks (GDM vs control group: 32.13 ± 1.47 vs 32.93 ± 1.47, *p* < 0.0001). At 32 weeks, fetuses in the GDM group had a higher AAWT after adjustment for possible confounders, as described above (Table 2). HC and FL remained lower in the GDM group after adjustment for maternal age, BMI, parity, fetal sex and gestational age at scan. The differences in HC and FL were lost when GWG was included in the model.

### Sex-specific changes of AAWT

Upon performing the analysis separately in male and female fetuses, the difference in AAWT between GDM and controls persisted for both sexes after adjustment for maternal age, BMI, parity, GWG and gestational age at 20 weeks (β coefficient 0.287 [95% CI 0.124, 0.450], *p* = 0.001 for female fetuses; β coefficient 0.220 [95% CI 0.061, 0.381], *p* = 0.007 for male fetuses) and 32 weeks (β coefficient 0.347 [95% CI 0.083, 0.610], *p* = 0.01 for female fetuses; β coefficient 0.639 [95% CI 0.406, 0.871], *p* < 0.0001 for male fetuses).

### AAWT and glycaemia at OGTT

The mean gestation at OGTT was 24.7 ± 2.45 weeks (GDM vs control group: 27.31 ± 3.10 vs 25.87 ± 3.08, *p* < 0.0001). There was an independent relationship between glycaemia and fetal adiposity. At 20 and 32 weeks, the FPG level at OGTT was significantly associated with AAWT after adjustment for maternal age, BMI, parity, GWG, fetal sex and gestational age at scan (20 weeks: β coefficient 0.195 [95% CI 0.120, 0.270], *p* < 0.0001; 32 weeks: β coefficient 0.233 [95% CI 0.111, 0.355], *p* < 0.0001). Neither 1hPG nor 2hPG levels were independently related to fetal abdominal adiposity.

## Discussion

Our results provide novel evidence that higher fetal adiposity is associated with GDM from as early as 20 weeks of gestation, a mean of 4.7 weeks before the biochemical diagnosis of GDM, in a South Asian population. The higher fetal adiposity at 20 weeks persisted after adjustment for maternal age, BMI, parity, GWG, fetal sex and gestational age at scan. The current literature on fetal adiposity in GDM reports a higher AAWT only in the third trimester, that is, well after 26 weeks of gestation and after the diagnosis of GDM [10–16]. To our knowledge, this is the first report of adiposity in early fetal life at 20 weeks. The increase in AAWT observed at 20 weeks persisted even at 32 weeks of gestation despite treatment for GDM.

An important point to note is that the excess adiposity was observed despite a smaller lean fetal mass (smaller AC, HC, FL and BPD at 20 weeks), signifying a disproportionate increase in adipose tissue over lean body mass. The disproportion between lean fetal mass and adiposity persisted at 32 weeks, with fetuses in the GDM group having a similar AC, HC, FL and BPD but higher AAWT.

In line with previous reports, women with GDM had significantly less GWG than controls [25]. After addition of GWG to the model, the differences in measures of lean fetal mass between GDM and controls at 32 weeks were lost, but differences in fetal adiposity persisted. These findings highlight the differential effects of GWG on fetal growth of lean and adipose tissue. With progressing gestation from 20 to 32 weeks, the difference in adiposity between GDM and controls increased, but the difference in measures of lean fetal mass became insignificant (Table 2), indicating the preferential growth of adipose tissue in the fetuses of women with GDM.

At birth, fetuses did not differ between groups with respect to birthweight or the proportion of LGA or SGA, again emphasising that increased fetal adiposity was observed in GDM despite a similar overall size. Most other studies that reported increased fetal adiposity in GDM also reported a higher offspring birthweight (ESM Table 1) [10, 12–14, 16]. It could therefore be argued that increased fetal adiposity observed in these studies was in fact a consequence of an overall increased size in GDM. Our results of increased fetal adiposity but similar fetal and neonatal size signify a preferential increase in adiposity over the growth of lean body mass in GDM. Catalano et al reported a similar disproportionate increase in fat mass compared with fat-free mass at birth in neonates of mothers with GDM [10]. Our study extends this finding to early fetal life at 20 weeks.

It is possible that the disproportionate fetal growth of adipose tissue over lean body mass is a prominent feature of glucose intolerance in South Asians. South Asian neonates are known to have a relatively high body fat and low lean mass for a given body weight hence the name ‘thin–fat’ babies [26]. We postulate that this differential growth process starts in early fetal life and is aggravated by GDM. The presence of other non-glycaemic factors such as dyslipidaemia, which is known to be higher in South Asians [27] and in GDM [28], could also contribute to higher fetal adiposity in GDM in South Asians [29].

We also observed that fetal adiposity was closely related to the maternal FPG level at OGTT at 24.7 weeks. This relationship between maternal FPG and adiposity has been reported at 28 and 37 weeks in women with normal glucose tolerance [30]. Catalano et al reported an independent relationship between maternal FPG level at OGTT and adiposity at birth in neonates of mothers with GDM [10]. Our study extends this association to early fetal adiposity at 20 weeks, prior to the diagnosis of GDM.

Our study had important limitations. It was retrospective and restricted to an urban, middle class southern Indian population. Detailed information on treatment of GDM and glycaemic control, which affects late pregnancy fetal growth, was not available. While treatment of GDM might have influenced the results of the 32 weeks, it would not affect our findings at 20 weeks. However, glucose measurement at the time of the 20 week scan would have provided more insight into the relationship between AAWT and maternal FPG level. A detailed dietary history, such as vegetarianism and total daily protein intake, which could in turn affect fetal growth, was not available. However, since this was a homogeneous cohort of affluent urban women from a single centre, the differences between groups are assumed to be small.

The key strength of our study is the availability of AAWT data at 20 weeks. AAWT can be measured easily from the routine 20 week anomaly scan carried out as a standard procedure across the world. The AAWT calculation uses data from the plane that is routinely captured for calculating AC. Thus, AAWT could serve as a potential marker to stratify risk and allow early biochemical testing for GDM in the subgroup of women with increased fetal adiposity. However, the potential of fetal adiposity measures for diagnosis and risk stratification needs to be studied in larger, prospective studies.

In summary, our study extends previous observations of fetal and neonatal adiposity in GDM to early fetal life at 20 weeks, pre-dating the biochemical diagnosis of GDM, and to a high-risk South Asian ethnic minority group. Our evidence highlights a need for more research into early fetal composition and adiposity in GDM, in larger prospective studies and other ethnic populations.

## Electronic supplementary material

Below is the link to the electronic supplementary material.

ESM 1 (PDF 58.5 kb)

## Abbreviations

1hPG: 1 h plasma glucose
2hPG: 2 h plasma glucose
AAWT: Anterior abdominal wall thickness
AC: Abdominal circumference
BPD: Biparietal diameter
FL: Femur length
FPG: Fasting plasma glucose
GDM: Gestational diabetes mellitus
GWG: Gestational weight gain
HC: Head circumference
LGA: Large for gestational age
SGA: Small for gestational age
