# Supplementary material for: Increased fetal adiposity prior to diagnosis of gestational diabetes in South Asians: more evidence for the ‘thin–fat’ baby
Source: Diabetologia. 2016 Dec 2;60(3):399–405. doi: 10.1007/s00125-016-4166-2 (PMC6518087; doi:10.1007/s00125-016-4166-2)
Supplement: Supplementary file 1 — (PDF 58.5 kb) [file 125_2016_4166_MOESM1_ESM.pdf]

Supplementary Table 1: Summary of the literature examining fetal biometry and fat mass in GDM

Study	Sample size	Gestational weeks	Adiposity measures	Other fetal biometry	Birth Weight (BW)	GDM diagnostic criteria
Larciprete [1]	85 GDM,<br>218 controls	Enrolled at 20-22 weeks	No difference seen prior to 31 weeks. Abdominal fat mass higher at 39-40 weeks, Mid-arm fat and supra-scapular fat mass higher at 31 weeks, mid-thigh fat higher at 37 weeks in GDM	Mid-thigh lean mass higher at 20-22 weeks in GDM	Higher in GDM	National diabetes data group (NDDG)
Aksoy [2]	55 GDM,<br>69 controls	26-28 weeks	AAWT: 4.07 ± 0.46 vs. 3.28 ± 0.37 mm (GDM vs. controls), p<0.0001	No difference	Higher in GDM	IADPSG
Tantanasis [3]	20 GDM,<br>15 control	24 and 26 weeks	Increased Subcutaneous fat at Abdomen GDM: 6.57 (0.99) vs. controls: 3.39 (0.61) mm, *p<0.0005*	Not reported	Not reported	(FPG: 7 mmol/l & 2hPG: 11.1 mmol/l)
De-Santis[4]	171 GDM,<br>43 controls	20-38 week serial measurements (only 15 scans <22 weeks)	Significantly higher fat mass (abdominal, supra-scapular, arm and thigh fat)	HC and BPD similar;<br>Faster growth of AC and FL in GDM	Higher BW z score in GDM, similar length	Carpenter Coustan
Vedavathi [5]	30 GDM,<br>30 controls	32-40 weeks	Not Studied	Higher AC and HC in GDM	Higher in GDM	Carpenter Coustan
Hammoud [6]	99 GDM,<br>145 Controls	17 and 37 weeks	Not studied	Similar AC, HC, FL	Not reported	100 g OGTT American Diabetes Association (ADA)
Sovio [7]	171 GDM,<br>3898 controls	20 and 28 weeks	Not Studied	2.0-fold risk of AC >90th centile, 1.52.0-fold risk of HC:AC ratio >10th centile at 28 weeks in GDM.<br>No difference at 20 weeks	Higher BW z score in GDM	WHO-1999 and IADPSG
Enzi [8]	17 GDM,<br>17 controls	At birth	% body fat: GDM (17% ± 1.7%) vs. controls (12.2% ± 0.5%) (newborn anthropometry)	Not reported	No difference	Whites Classification
Catalano [9]	195 GDM,<br>220 controls	At birth	Restricting comparisons between infants appropriate for gestational age with GDM and controls: Fat mass in g: (371 ± 163 g vs. 329 ± 150 g, p = 0.002) (GDM vs. controls): Body fat (%): 11.4% ± 4.6% vs. 9.9% ± 4.0%, p = 0.002 (GDM vs. controls).	No difference in lean mass, HC, leg length, AC. (Whole GDM group vs. controls)	Higher proportion of LGA in GDM group.	NDDG
Nasrat [10]	51 GDM,<br>501 controls	At birth	Increased skim fold: Biceps, subscapular, supra-iliac, sum of all skin folds.	Similar HC and AC	Higher in GDM	ADA

[1] Larciprete G, Valensise H, Vasapollo B, et al. (2003) Fetal subcutaneous tissue thickness (SCTT) in healthy and gestational diabetic pregnancies. Ultrasound Obstet Gynecol 22: 591-597
[2] Aksoy H, Aksoy U, Yucel B, Saygi Ozyurt S, Aydin T, Alparslan Babayigit M (2015) Fetal anterior abdominal wall thickness may be an early ultrasonographic sign of gestational diabetes mellitus. The journal of maternal-fetal & neonatal medicine : the official journal of the European Association of Perinatal Medicine, the Federation of Asia and Oceania Perinatal Societies, the International Society of Perinatal Obstet: 1-5
[3] Tantanasis T, Daniilidis A, Giannoulis C, et al. (2010) Sonographic assessment of fetal subcutaneous fat tissue thickness as an indicator of gestational diabetes. European journal of obstetrics, gynecology, and reproductive biology 152: 157-162
[4] de Santis MS, Taricco E, Radaelli T, et al. (2010) Growth of fetal lean mass and fetal fat mass in gestational diabetes. Ultrasound Obstet Gynecol 36: 328-337
[5] Vedavathi KJ SR, Kanavi Roop Shekharappa , Venkatesh G , Veerananna HB (2011) Influence of Gestational Diabetes Mellitus on Fetal growth parameters. Int J Biol Med Res 2: 832-834
[6] Hammoud NM, Visser GH, Peters SA, Graatsma EM, Pistorius L, de Valk HW (2013) Fetal growth profiles of macrosomic and non-macrosomic infants of women with pregestational or gestational diabetes. Ultrasound Obstet Gynecol 41: 390-397
[7] Sovio U, Murphy HR, Smith GC (2016) Accelerated Fetal Growth Prior to Diagnosis of Gestational Diabetes Mellitus: A Prospective Cohort Study of Nulliparous Women. Diabetes Care 39: 982-987
[8] Enzi G, Inelmen EM, Caretta F, Villani F, Zanardo V, DeBiasi F (1980) Development of adipose tissue in newborns of gestational-diabetic and insulin-dependent diabetic mothers. Diabetes 29: 100-104
[9] Catalano PM, Thomas A, Huston-Presley L, Amini SB (2003) Increased fetal adiposity: a very sensitive marker of abnormal in utero development. Am J Obstet Gynecol 189: 1698-1704
[10] Nasrat H, Abalkhail B, Fageeh W, Shabat A, el Zahrany F (1997) Anthropometric measurement of newborns of gestational diabetic mothers: does it indicate disproportionate fetal growth? J Matern Fetal Med 6: 291-295
